# A Microstructured Fiber with Defined Borosilicate Regions to Produce a Radial Micronozzle Array for Nanoelectrospray Ionization

**DOI:** 10.1038/srep21279

**Published:** 2016-02-19

**Authors:** Y. Fu, S. Morency, K. Bachus, D. Simon, T. Hutama, G. T. T. Gibson, Y. Messaddeq, R. D. Oleschuk

**Affiliations:** 1 Department of Chemistry, Queen’s University, Kingston, ON, K7L 3N6, Canada; 2 Centre d’optique, photonique et laser (COPL), Quebec city, QC, G1V 0A6, Canada

## Abstract

This work highlights the possibility of using microstructured fibres with predefined doped regions to produce functional microstructures at a fibre facet with differential chemical etching. A specially designed silica microstructured fibre (MSF) that possesses specific boron-doped silica regions was fabricated for the purpose of generating a radial micronozzle array. The MSF was drawn from a preform comprising pure silica capillaries surrounded by boron-doped silica rods. Different etching rates of the boron-doped and silica regions at the fiber facet produces raised nozzles where the silica capillaries were placed. Fabrication parameters were explored in relation to the fidelity and protrusion length of the nozzle. Using etching alone, the nozzle protrusion length was limited, and the inner diameter of the channels in the array is expanded. However with the addition of a protective water counter flow, nozzle protrusion is increased to 60 μm with a limited increase in hole diameter. The radial micronozzle array generated nine individual electrosprays which were characterized using spray current measurements and related to theoretical prediction. Signal enhancement for the higher charge state ions for two peptides showed a substantial signal enhancement compared to conventional emitter technology.

Microstructured fibers (MSFs) represent a class of optical fiber that features an array of air channels as part of a cladding designed to guide light through the core by total internal reflectance^1^. Typically, these fibers are composed of silica and the channels are evenly spaced and homogeneous in diameter. The construction of MSFs starts with a preform at a manageable scale, either a disc through which holes are drilled or, more often, an assembly of tubes and rods, where the holes/tubes are carefully placed according to a design. This preform is then drawn, sometimes in steps, at high temperatures to a thin fiber that retains the pattern of the preform^2^.

Often, silica MSFs contain regions of doped silica, as doped regions are known to cause the fiber to become birefringent due to stress applied by differences in thermal expansion^3^,^4^. Such MSFs are typically fabricated in the same “stack and draw” manner as conventional silica MSFs, but the preform tubes and rods are not all the same material. An example is the polarization-maintaining silica MSF in Figure S1A, which contains doped silica on either side of the core.

Wet chemical etching, particularly by hydrofluoric acid (HF), is a commonly used approach to remove silica in a predictable and well-controlled manner. It is well known that different types of silica are etched by HF at different rates, which allows the fabrication of structure at the tip of a MSF after etching if that fiber is comprised of more than one type of silica^5^,^6^,^7^. Selective etching of these doped regions is demonstrated in Figure S1B,C, where HF etches these regions faster than silica to leave depressions and ammonium bifluoride (AF), another commonly used etchant, etches these regions slower than silica to leave raised plateaus. These etchants are in an equilibrium in aqueous solution depending on pH and F^−^ concentration, giving rise to a conditional difference in etching selectivity. With the versatility imparted in the patterning of MSFs by preform drawing methods, coupled with the flexibility in etching selectivity of various doped silica materials and with etchant pH, the shape, size, and pattern of surface structures is extremely variable and precisely controlled.

One application of this approach to materials fabrication is the development of multiplexed emitters for electrospray ionization (ESI). In ESI mass spectrometry, the effects of multiple electrospray (MES) impart a major advantage in the analysis of ions, particularly for biological samples such as proteins^8^. Much of the benefit is associated with the dependence of ESI on solution flow rate. As the flow rate drops, especially to the nL min^−1^ range, the charged droplets that are ejected from the electrospray plume become smaller and the efficiency of charge transfer to analyte molecules in solution improves^9^. Popular commercial emitters for these lower flow rates are fused-silica capillaries that have been pulled to a fine, tapered exit in order to support stable cone-jet (Taylor cone) electrospray. However, these single-channel tapered emitters are limited in the range of flow rates they can use.

To overcome these issues, larger flow rates may be split into *n* individual electrosprays, each one offering the benefit of much lower-flow electrospray. The theoretical relationship between electrospray current (*I*_*total*_) at a given flow rate and the electrospray current (*I*_*s*_) of the same flow split into *n* individual emitters has been found to be 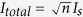
10, meaning that the detection signal can be enhanced by a factor of 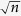 by having an emitter with *n* separate spraying tips. A variety of emitters have been developed using this approach^11^, including examples fabricated by microchip fabrication techniques, laser ablation, or simply assembling an array of conventional fused-silica capillary-based emitters^10^,^12^. These emitters are typically large and do not couple well with traditional MS inlets^12^,^13^, are complicated to fabricate^14^,^15^, or usually both^10^,^16^.

Microstructured fibers present an interesting alternative approach to MES emitters, as they can be fabricated to any design having dimensions compatible with conventional LC and MS equipment. However, commercially available MSFs have channels that are too close together to enable MES^17^. Polycarbonate MSFs (lacking individual nozzles) generated MES with fully aqueous samples only, due to wetting effects^18^. Alternatively, a custom silica MSF with polymeric nozzles produced MES however the emitters were fragile^19^. In this work, MSFs were designed to have channels made from a different preform material than the bulk of the fiber, such that selective etching could be used to make a nozzle at each channel exit. A drawing of the design appears as Fig. 1A. In the design, nine channels were set 100 μm apart to prevent interference between electrosprays, and arranged in a radial pattern to ensure electrical shielding effects are equivalent for all channels^13^. The outer diameter (o.d.) of the fiber was 360 μm to match standard fused-silica capillary, for which polyether ether ketone (PEEK) fittings are commercially available. The nine capillaries that form the channels are made of pure silica while the large center rod and the small rods that fill the space around the nine silica capillaries are doped by 9 mol% boron (shaded regions shown in Fig. 1A). Because these doped regions are etched faster than pure silica by HF, the silica immediately surrounding the channels protrudes from the fiber tip following etching to form micronozzles^20^. Channel diameter is 10 μm, which is similar to the aperture diameter of standard tapered nanoESI emitters.

The design was assembled into the preform in Fig. 1B, which was drawn into the fiber shown in Fig. 1C,D. The SEM image (D) shows the simple surface structure of the cross section of the MSF, but the optical image (C) shows the nature of the silica composition due to the different refractive indexes of the pure silica and boron-doped regions, which appear darker. From this image it can be seen that the boron-doped silica rods lose their shape and fill in the space around the pure silica tubes, owing to the lower temperature at which the doped silica melts.

After etching the MSF tip with HF, which selectively etches the doped regions faster, the optical image in Fig. 1C gives a good indication of what the final structure will look like and where the nozzles will appear. SEM imaging was used to determine the degree of nozzle protrusion and the channel diameter altered by the etching procedure. A series of fibers were etched in the HF solution for various times from 6 to 17 minutes, with representative SEM images shown in Fig. 2.

As the fiber is chemically etched, the pure silica regions are etched at the same rate over the face of the fiber tip, while the boron-doped regions are etched more quickly and occupy a deeper plane. The silica features, such as the rims defined by the inner and outer silica tubes in the design, are also etched laterally as etchant is able to access the sides of these features after the surrounding borosilicate is removed. As etching continues, lateral etching eventually removes all the silica features at the outside of the MSF and channels are no longer enclosed. An important consequence of this lateral etching is the widening of the channels as etchant enters the tubes and etches the silica there. The diameter of the channels increased to 29 ± 0.5 μm from 8.2 μm after only 6 minutes of etching. From these measurements, the etch rate of the pure silica parts is calculated to be 1.7 μm min^−1^.

In order to prevent the etchant from entering the channels and widening the openings, water was flowed through the channels from the opposite end at a linear velocity that overcomes the diffusion of HF into the channels^21^. Ultimately, this approach will dilute the etchant near the tip surface, so the lowest possible flow rate should be used. The total water flow rate was found to give reproducibly negligible channel widening at 75 nL min^−1^, which was used for all further etching.

The etching time for MSFs immersed in HF etchant was optimized for nozzle shape and protrusion length. As etching time increases, the lateral etching of the silica tubes that form the nozzles causes the silica surrounding the channels to be removed. Etching time, therefore, is essentially limited by the time it takes for the loss of silica to reach the channel. This etching time was found to be 14 min for the fiber in this work (Fig. 1), where the silica remaining around a given channel is only ~10 μm thick, ultimately defining the nozzle tip. Presented in Fig. 3A and B are SEM images showing the front and side views of a MSF face after etching for 14 min in HF with 75 nL min^−1^ total water counter flow. The nozzle protrusion length, defined as the distance from the tip of the nozzle to the bottom plateau comprised of borosilicate glass, was found to be 60.8 ± 1.2 μm. This translates to borosilicate parts etching 4.3 μm min^−1^ faster than silica parts, or about 6.0 μm min^−1^ (after adding 1.7 μm min^−1^ calculated above for the silica parts).

The effectiveness of the multinozzle MSF as a MES emitter was tested using electrospray current measurements while visually monitoring the tip to ensure the electrospray maintained a stable cone-jet at all nozzles. A microscope photo exhibiting stable, independent electrosprays in cone-jet mode coming from each nozzle is shown in Fig. 3D. Video VS1 of the Supporting Information shows this emitter in operation, with the plane of focus changing to allow clear visualization of all nine individual Taylor cones.

Further testing revealed that the emitters support MES over a wide range of operating conditions. Nine individual electrosprays were observed (Figure S2) for applied voltages of 2.2–3.4 kV (at 0.3 μL min^−1^), and (Figure S3) for total flow rates from 100 nL min^−1^ to 3.0 μL min^−1^ (at 2.8 kV). The small electric current generated by the electrospray was measured for all conditions tested, with the value for a particular condition being the mean current over at least 5 minutes acquisition time and the uncertainty being the standard deviation of the mean. The dependence of spray current on total flow rate and voltage followed the expected trends as previously observed^19^. The solvent composition was varied according to a typical LC/MS gradient, from 99:1 water:methanol (by volume, with 1% acetic acid) to 50:50 water:methanol and back, as shown in Figure S4 of the Supporting Information. The spray current followed the solvent composition well, without a significant change in current when the composition returned to a similar value. More interesting, however, is the dependence of electrospray current on the number of nozzles (*n*). Such a plot, shown in Fig. 4A, may be obtained by measuring the current for 9-nozzle emitters where some of the nozzles have been blocked at the inlet. The linearity of the dependence of current on 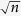 (linear regression shows R^2^ = 0.99) is indicative of true MES behavior. This plot demonstrates the greatest advantage of MES emitters, that the ion signal is enhanced by a factor of 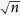 relative to that of a single-channel emitter at the same total flow rate. In addition to spray current, the intensities of individual analyte ions can be monitored to examine the enhancement afforded by a multispray emitter. Two model peptides, which are representative of proteomic analytes (angiotensin and bradykinin; monitored for the ions (M+2H^+^)^2+^ = 523.77, (M+3H^+^)^3+^ = 349.52 and (M+2H^+^)^2+^ = 530.79, (M+3H^+^)^3+^ = 354.19 respectively) showed a significant ion count enhancement when using the MES emitter. Figure 4B shows the ion counts obtained by infusing a 10 μM solution of each peptide at two flow rates, 300 and 900 nL min^−1^. The MES emitter produces a signal enhancement compared to a conventional single tapered fused-silica emitter for each of the analytes and charge states, at each of the flow rates tested. The enhancement for the lower charge state ions (i.e. (M+2H^+^)^2+^) was limited for angiotensin and higher for bradykinin with relative increases in ion counts per second of 1.16 and 3.72 respectively. However, the signal enhancement for the higher charge state ions (i.e. (M+3H^+^)^3+^) for both peptides showed a substantial increase of 16 and 35 times signal enhancement compared to the current research standard. The multinozzle MSF designed and fabricated in this work was thus demonstrated to be an effective MES emitter, useful for enhancing the ion signal of analyses using electrospray, particularly ESI-MS. This approach offers an elegant and versatile way of solving a complicated fabrication problem, especially for LC/MS for which it can be used as a MES emitter having full dimensional compatibility with conventional LC and MS equipment. Although the custom doped fiber was applied to the fabrication of MES emitters in this work, the methodology is applicable not only to other electrospray applications, but to any instance where microstructure at the fiber facet is important (e.g. optical coupling or lensing).

## Methods section

### MSF fabrication

The MSF was fabricated at Canada Excellence Research Chair in Photonics Innovations (CERCP) (Québec City, Canada). A large borosilicate rod was inserted into a large thin-walled silica capillary. Surrounding this were 9 silica capillaries and 9 borosilicate glass rods, with 36 small filler rods among them, which together was inserted into an outer silica tube. The arrangement and diameters of the tubes and rods were chosen by CERCP based on experience such that the target dimensions will be met upon drawing (Figure S5: photo of drawing tower used for fiber fabrication). The dimensions of the drawn MSF were measured to be: o.d. of the fiber is 363.7 ± 0.8 μm, channel diameter is 8.2 ± 0.1 μm and the pitch of the channels is 96.3 ± 1.0 μm residing on a centered circle with a diameter of 281.5 ± 2.0 μm.

### Emitter preparation

Prior to etching, part of the MSF’s protective polyacrylate coating was removed thermally using a wire stripper (Stripall TWC-1, Teledyne Impulse, San Diego, CA, USA). A fiber cleaver (LDC-400, Vytran, Morganville, NJ, USA) was then used in the stripped area to produce a straight cleave for etching. The fiber was connected to a syringe filled with deionized water via PEEK fittings (IDEX, PK-120BLK with a F-185x sleeve). A syringe pump (Harvard Apparatus Pump 11 Plus, Holliston, MA, USA) was used to control the rate of flow. The other end was observed under an optical microscope (Nikon Eclipse ME600, Nikon Canada, Mississauga, Canada) or a USB microscope (Veho VMS-004D) to ensure all 9 channels had water flowing at the desired rate. After confirmation of flow, the etching end was placed into a 1.5 mL microcentrifuge tube containing 350 μL of 48 wt% HF (aq) such that the tip of the MSF was suspended in etchant. The fiber was etched with constant flow of 75 nL min^−1^ water for 14 minutes in a fumehood (22 ± 1 ^o^C), and then transferred to a microcentrifuge tube containing water to quench further etching and clean the tip of debris. The fiber was left in the water with a high flow rate of flushing water for 25 minutes. The etched fiber was cleaved at a length of 6–7 cm, and the remaining protective coating was removed to give a final emitter, which was examined by a microscope to determine if all nozzles were generated evenly by the etching process. A schematic of the apparatus is shown in Figure S6 of the Supporting Information. *Note: HF solutions are hazardous and care should be taken to prevent exposure to HF liquid or vapor. HF solutions should be handled in a ventilated hood and protective equipment should be worn.*

To make the surface of the emitter less wetted by the electrospray solvent, it was chemically modified by a hydrophobic group through a silanization reaction^17^. After drying the emitters at 150 °C for >6 hours, the tip of an emitter was immersed into the silanization solution, comprising 3:1 (v/v) toluene:CTMS (totaling 400 μL), in the same manner as for etching. The emitters were left in the solution overnight, at which time the emitters were rinsed with acetonitrile (95% in water) using an HPLC pump for 20 minutes and stored in a desiccator until needed.

### Electrospray current measurement and imaging

The experimental setup for measuring the electrospray current and imaging the spray is similar to that used previously^19^ and is shown in Figure S7 of the Supporting Information. A solution composed of deionized water (79.2% v/v), methanol (19.8% v/v) and glacial acetic acid (1% v/v), degassed and filtered, was delivered to the emitter using a nano-flow gradient pump (IDEX Health & Science LLC, Oak Harbor, WA, USA). Voltage was applied via a PEEK micro-tee using a liquid junction. The emitter was held in place facing an aluminum block ground electrode, the tip being 2 ± 0.3 mm away. This entire assembly was placed on the stage of an inverted microscope (Nikon Eclipse Ti-S, Nikon Canada, Mississauga, Canada), with images and video being captured using a Nikon DS digital camera. The presence and number of stable Taylor cones was easily observed from a side view. Voltage for electrospray was supplied using a TriSep 2100 high-voltage module (Unimicro Technologies, Pleasanton, CA, USA), and a picoammeter (Model 6485, Keithley Instruments Inc., Cleveland, OH, USA) was used to measure the small current generated by the electrospray. Electrospray current was measured for 5 minutes for each run and the mean and standard deviation were used as the data point and uncertainty. Additionally, a solvent gradient from 1% methanol in water to 50% methanol and back was used to test the multi-nozzle emitters for their performance under typical LC conditions.

### Scanning electron microscopy (SEM)

Emitter tips were gold coated using a Hummer sputtering system (Anatech USA, Union City, CA, USA). SEM images were obtained using a FEI-MLA (Hillsboro, OR, USA) Quanta 650 Field Emission Gun-Environmental SEM.

### Online electrospray ionization mass spectrometry

Emitters were tested online using a Thermo Scientific LTQ Orbitrap Velos hybrid FT mass spectrometer (MS) with a nanoelectrospray source, connected via a liquid junction to a syringe pump, which delivered electrospray solution at a given flow rate, through a transferring capillary (360 μm o.d. and 75 μm i.d.). The voltage was set to 1.8 kV with a capillary temp of 275 °C. The scans were in the FTMS, which was tuned at a resolution of 60,000 at 400 m/z. The AGC time was set to 500 ms or 1.0 × 10^6^ ions over the 150–1200 m/z range. A 15 μm aperture tapered emitter (PicoTip) was utilized at 300 nL min^−1^ and a 30 μm aperture tip at 900 nL min^−1^ commensurate with manufacturer specifications for flow rate range. Raw spectra for angiotensin with both emitter types at 300 and 900 nL min^−1^ are shown in Figure S8.

## Additional Information

**How to cite this article**: Fu, Y. *et al.* A Microstructured Fiber with Defined Borosilicate Regions to Produce a Radial Micronozzle Array for Nanoelectrospray Ionization. *Sci. Rep.* **6**, 21279; doi: 10.1038/srep21279 (2016).

## Supplementary Material

Supplementary Information

Supplementary Video VS1

## Figures and Tables

**Figure 1:**
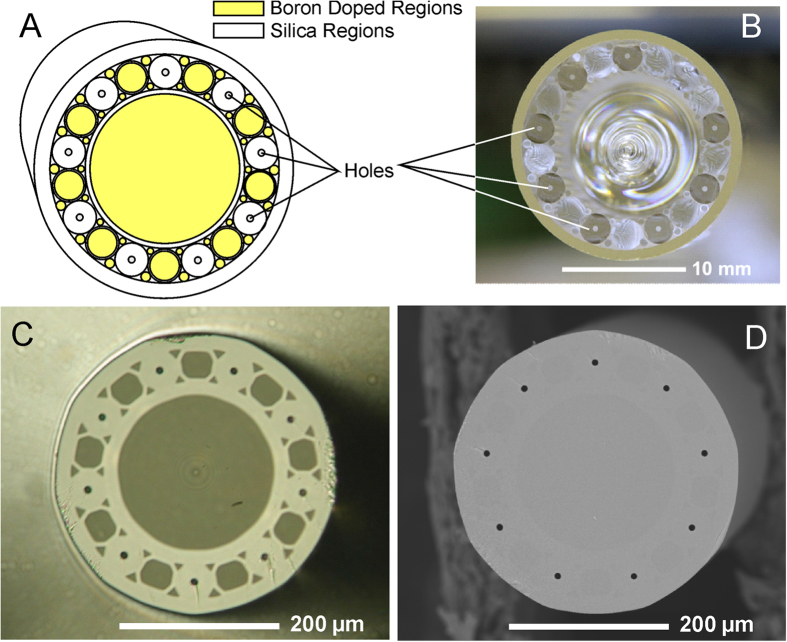
(**A**) Design for the silica MSF with boron-doped regions (9 mol% boron) having nine channels arranged in a radial array. (**B**) Optical image of the preform of the MSF with doped regions constructed by COPL. Scale bar is labeled on the image. (**C**) Optical and (**D**) SEM images of the drawn fiber showing nine channels in a radial array and boron-doped regions as darker areas.

**Figure 2:**
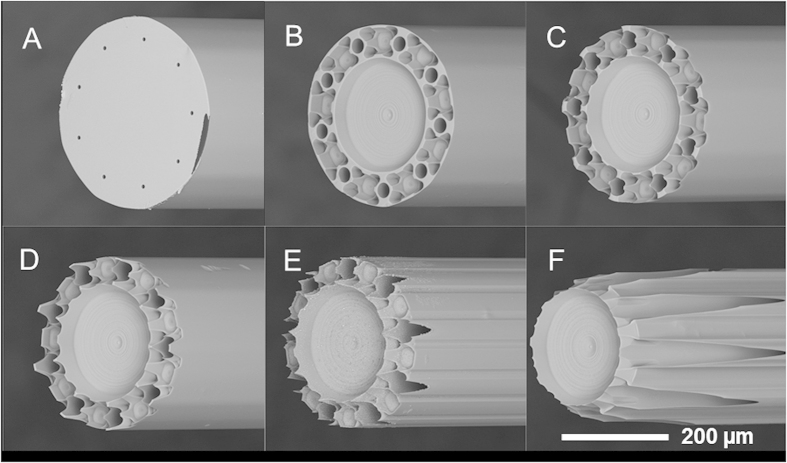
SEM images of a MSF (**A**) before etching, and after etching in HF for (**B**) 6 minutes, (**C**) 8 minutes, (**D**) 10 minutes, (**E**) 12 minutes and (**F**) 17 minutes. There was no water flowing through the fiber during etching.

**Figure 3:**
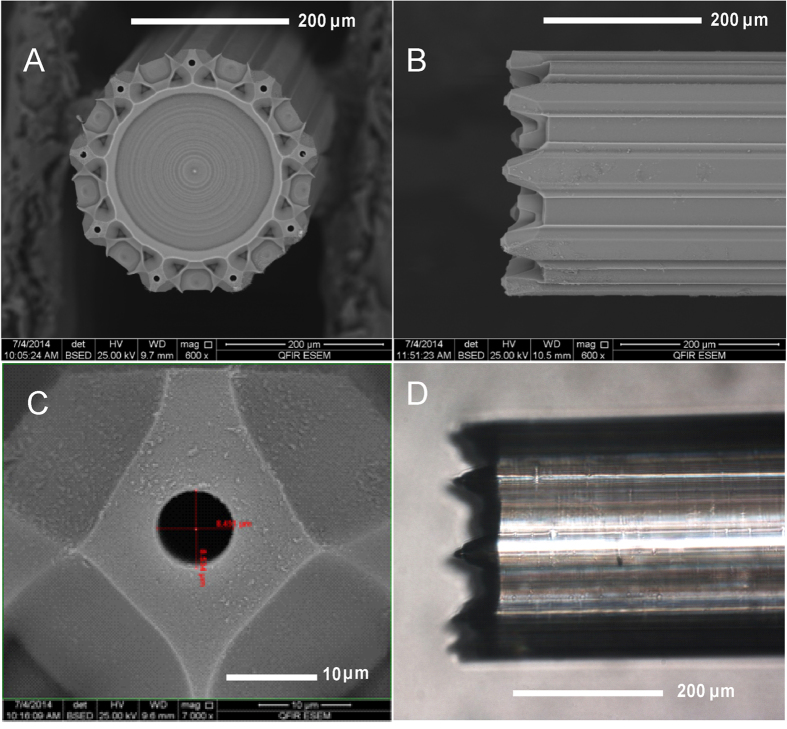
SEM images of (**A**) the top view and (**B**) the side view of a multi-nozzle MSF emitter generated by etching in HF for 14 minutes with a water flow rate of 75 nL min^−1^, having average nozzle protrusion length of 60.8 μm and channel diameter of 8.3 μm. (**C**) Magnified image of a single nozzle, top view. (**D**) Photomicrograph (200× magnification) of nine individual electrosprays (focused on the front two sprays) in stable cone-jet mode generated from the multi-nozzle emitter with hydrophobic coating (CTMS). Conditions: 79.8% water/19.2% methanol/1% acetic acid at 300 nL min^−1^ total flow rate, 2.8 kV applied potential and 2 mm working distance.

**Figure 4:**
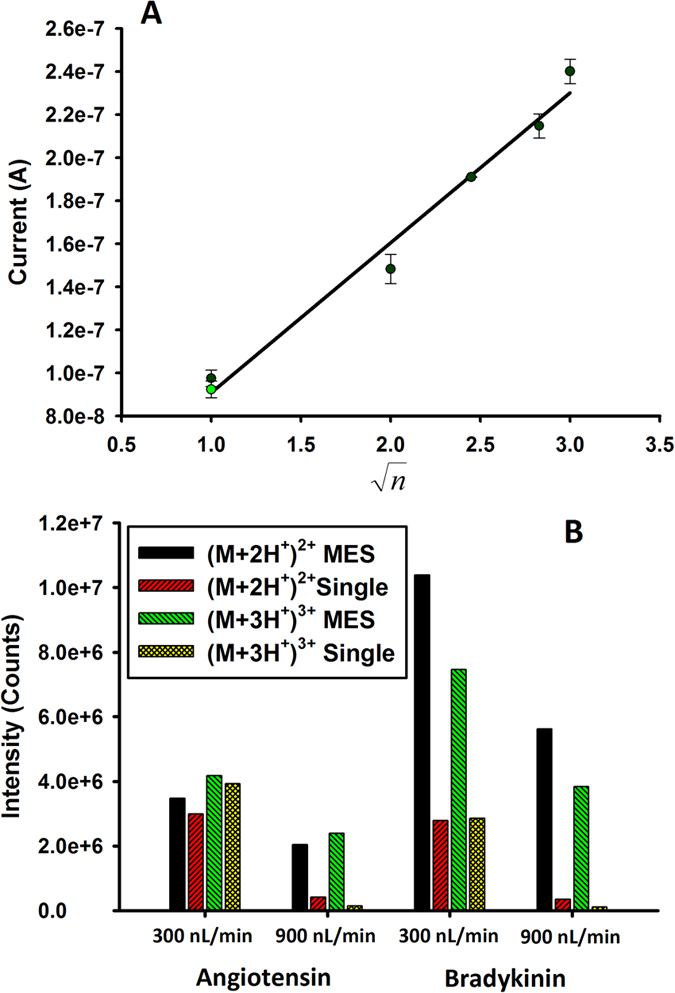
(**A**) Electrospray current as a function of the square root of the number of spraying nozzles, demonstrating MES behavior of the multinozzle MSF emitter. The two data points in the plot at n = 1 indicate the spray current produced by a 10 μm (black) and 8 μm i.d. (green) SilicaTip™ emitter, respectively, under the same conditions (applied potential is 1.2 kV) (B) Ion current enhancement observed for the MES emitter compared to a single tapered emitter at two flow rates (300 and 900 nL/min) for an angiotensin and bradykinin sample.
