# Supplementary material for: A Microstructured Fiber with Defined Borosilicate Regions to Produce a Radial Micronozzle Array for Nanoelectrospray Ionization
Source: Sci Rep. 2016 Feb 19;6:21279. doi: 10.1038/srep21279 (PMC4759573; doi:10.1038/srep21279)
Supplement: Supplementary Information [file srep21279-s1.doc]

**A Microstructured Fiber with Defined Borosilicate Regions to Produce a Radial Micronozzle Array for Nanoelectrospray Ionization**

*Yueqiao Fu, Steeve Morency, Kyle Bachus, David Simon, Timothy Hutama, Graham T.T. Gibson, Younès Messaddeq and Richard D. Oleschuk**

**Video SV-1.** Multi-nanoelectrospray from an HF-etched boron-doped microstructured fiber. Nine individual Taylor cones are visible as the microscope is adjusted for depth of focus to bring each cone into view. MES was operating at a flow rate of 300 nL min^-1^ with a 2.8 kV applied potential.


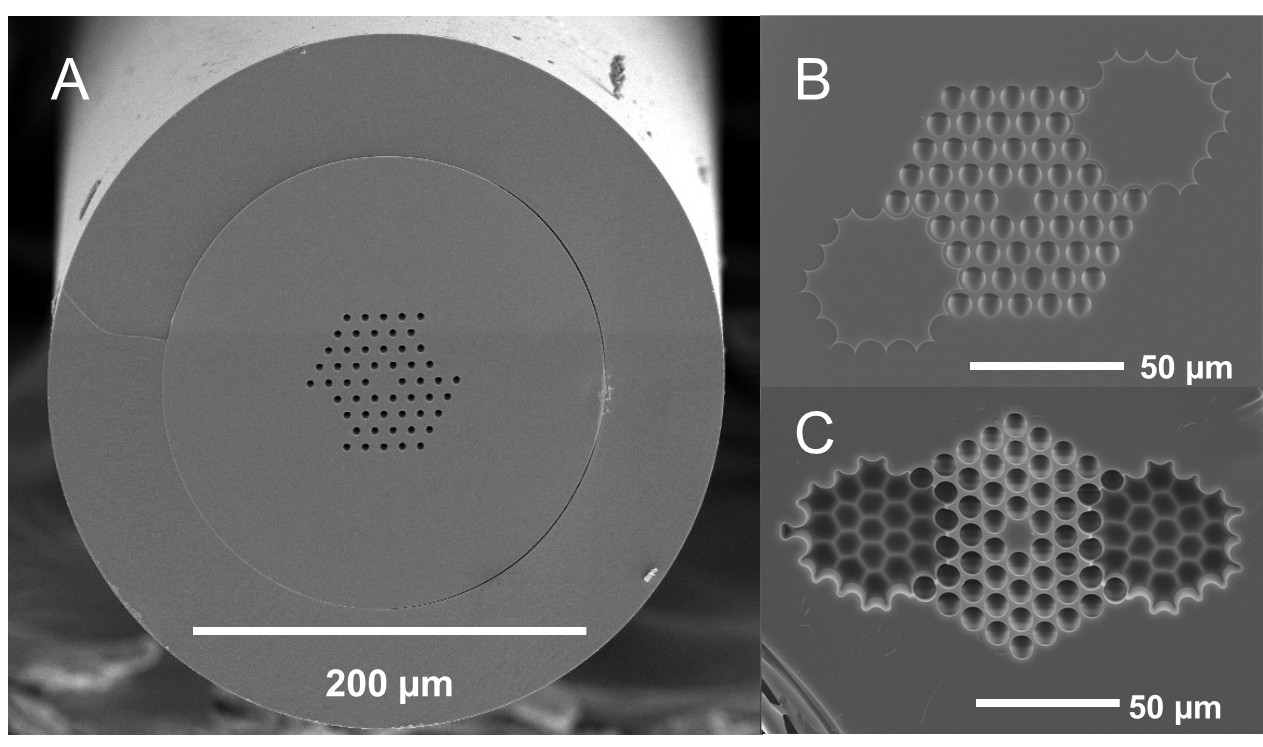


**Figure S1.** Scanning electron microscopy (SEM) images of the cross section of a MSF (a LMA-PM-15, NKT Photonics, Birkerød, Denmark) with 54 channels and stress-applying parts on either side of the channel region: (a) bare fiber with protective coating; (b) fiber after etching in AF for 12 minutes; (c) fiber after etching in HF for 1 minute.


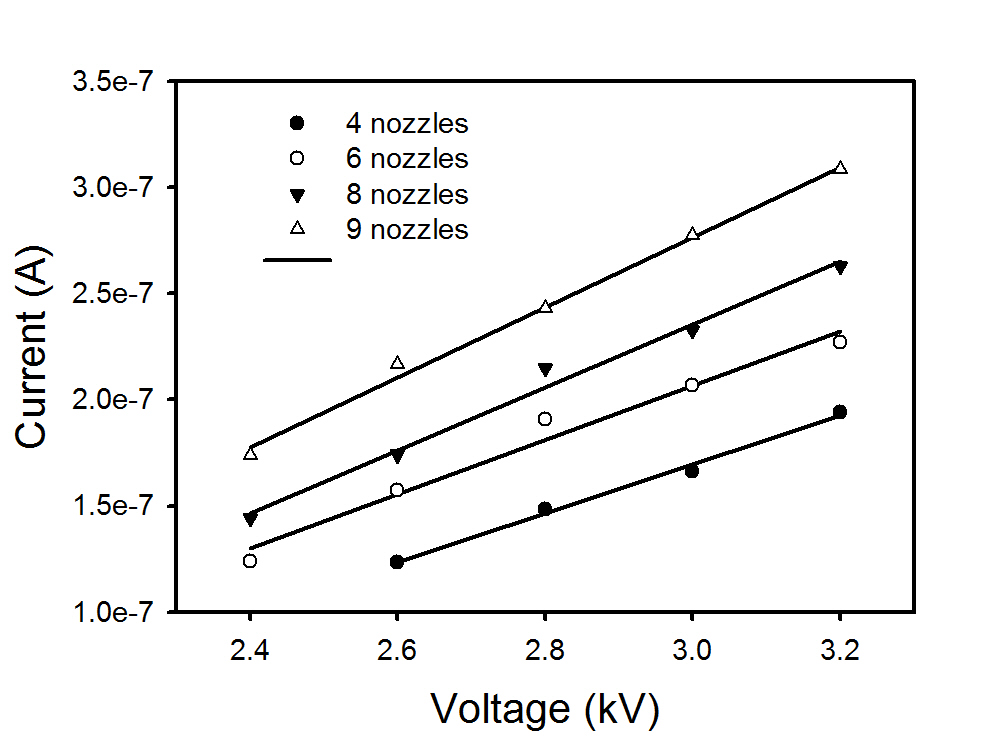
**Figure S2.** Spray current for a MES emitter over a Voltage range of 2.4-3.2 with different numbers of nozzles spraying. Conditions: 2.8 kV applied potential and 300 nL min^-1^ flow rate.


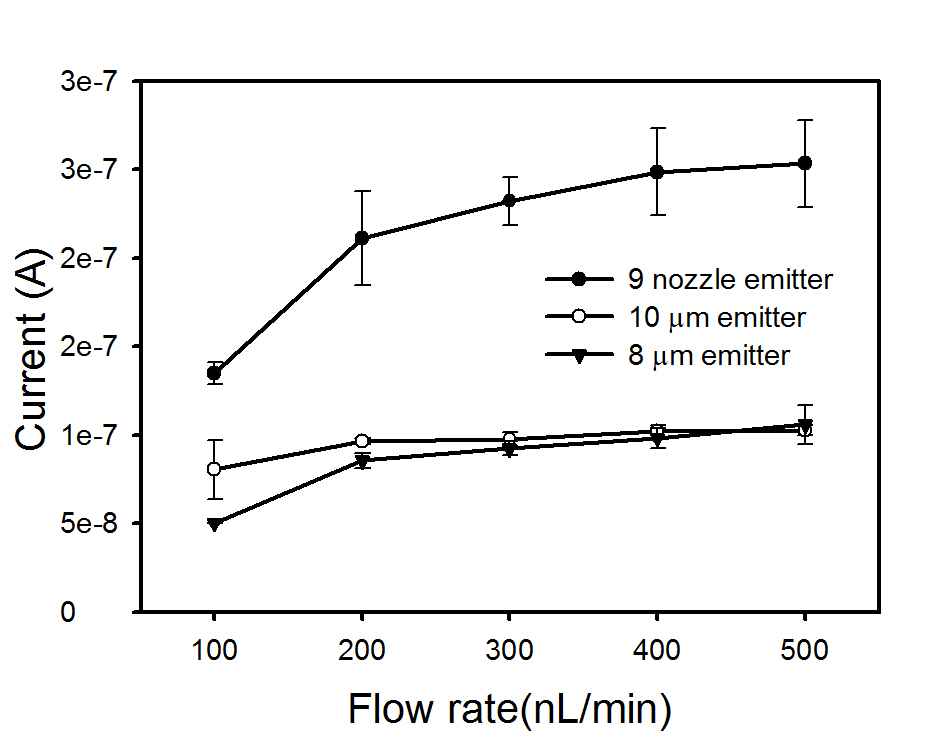
**Figure S3.** Spray current over a flow rate range of 100-500 nL min^-1^. Conditions: 2.8 kV applied potential and 300 nL min^-1^ flow rate.


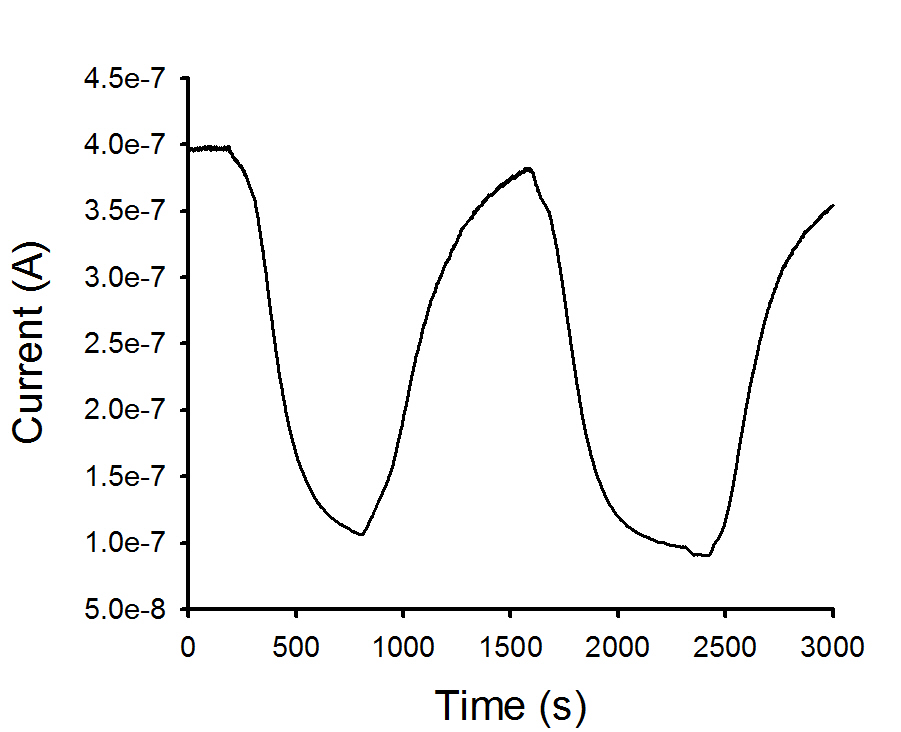


**Figure S4.** Spray current changes with solvent composition using a MES emitter. Following a typical solvent gradient encountered during LC/MS analysis, the emitter experienced two gradient cycles from 99:1 water:methanol to 50:50 water:methanol and back. The first 1 minute held the initial solvent composition. Conditions: 2.8 kV applied potential and 300 nL min^-1^ flow rate.

**Figure S5.** Fiber drawing tower used to pull the custom boron doped microstructured fiber at COPL.


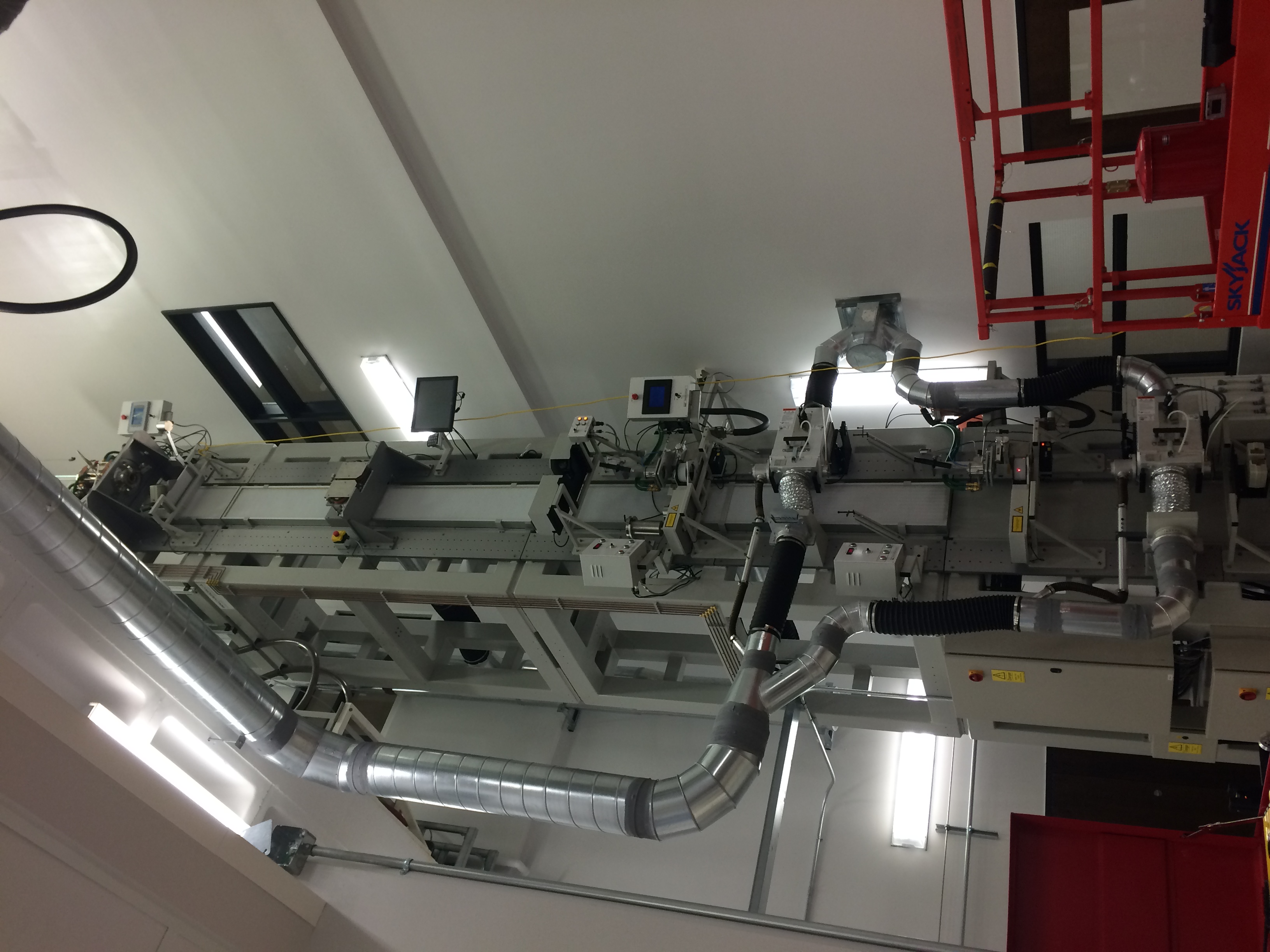


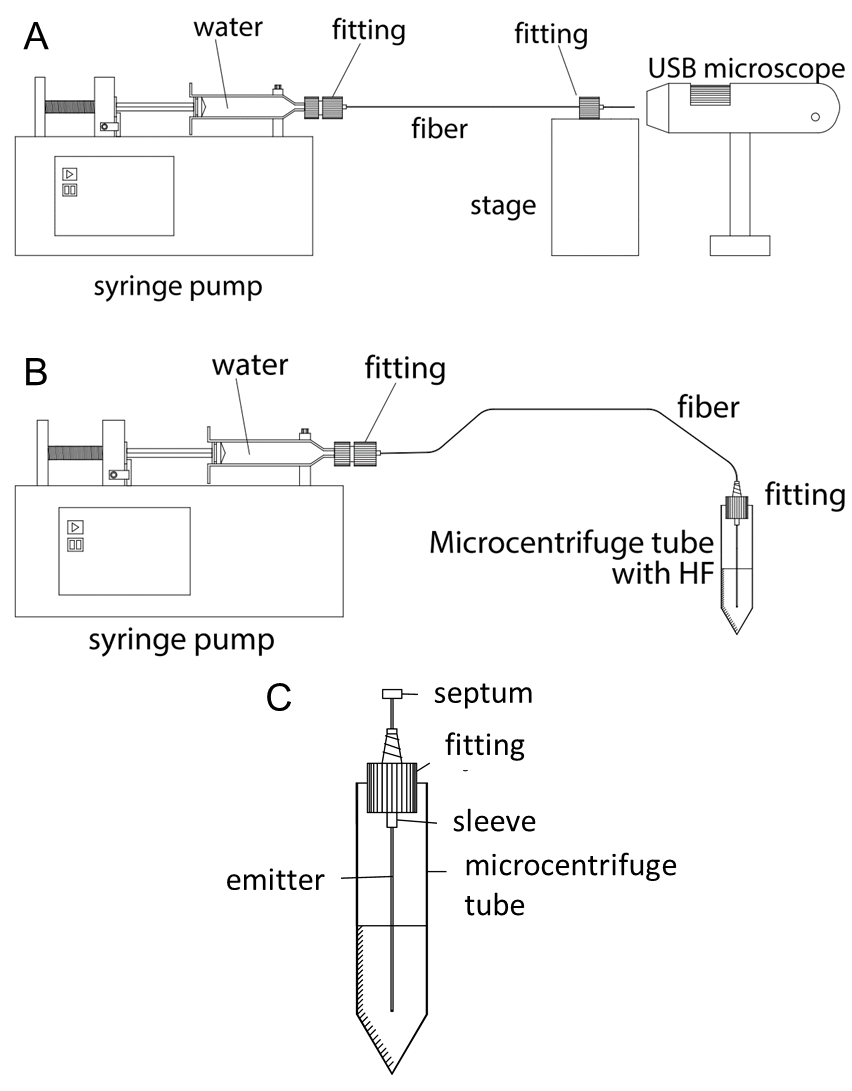


**Figure S6.** Schematic setups of (A) USB microscope and syringe for confirmation of water flow from all 9 channels of the MSF, (B) etching MSF end in concentrated HF with water flow and (C) silanization step showing immersion of etched tip of an emitter into the reagent solution.


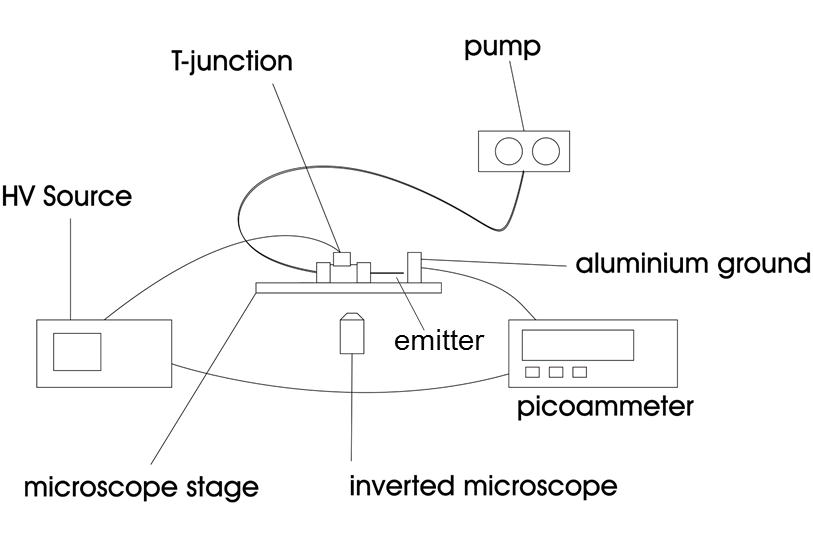


**Figure S7.** Schematic diagram of offline electrospray current measurement/spray imaging apparatus for electrospray testing.

# Angiotensin PicoTip – 300nL/min

# Angiotensin PicoTip – 900nL/min

# Angiotensin MES – 300nL/min

# Angiotensin MES – 900nL/min

**Figure S8.** Raw Mass Spectra obtained from a commercial tapered emitter at 300 (15 µm aperture tip) and 900 nL min^-1^ (30µm aperture tip) and the corresponding spectra obtained with a MES emitter at 300 and 900 nL min^-1^ for a 1 µM solution of Angiotensin.
